# Development of *Houttuynia cordata* Extract-Loaded Solid Lipid Nanoparticles for Oral Delivery: High Drug Loading Efficiency and Controlled Release

**DOI:** 10.3390/molecules22122215

**Published:** 2017-12-13

**Authors:** Ju-Heon Kim, Jong-Suep Baek, Jin-Kyu Park, Bong-Joo Lee, Min-Soo Kim, Sung-Joo Hwang, Jae-Young Lee, Cheong-Weon Cho

**Affiliations:** 1 College of Pharmacy and Institute of Drug Research and Development, Chungnam National University, 99 Daehak-ro, Yuseong-gu, Daejeon 34134, Korea; moom5611@naver.com (J.-H.K.); baekjs@cnu.ac.kr (J.-S.B.); jaeyoung@cnu.ac.kr (J.-Y.L.); 2 Wissen Co., Ltd., #410 Bio Venture Town, 461-8, Daejeon 305-811, Korea; jk4101@hanmail.net; 3 College of Veterinary Medicine, Chonnam National University, Gwangju 500-757, Korea; bjlee@chonnam.ac.kr; 4 College of Pharmacy, Pusan National University, 63 Busandaehak-ro, Geumjeong-gu, Busan 609-735, Korea; minsookim@pusan.ac.kr; 5 College of Pharmacy and Yonsei Institute of Pharmaceutical Sciences, Yonsei University, 162-1 Songdo-dong, Yeonsu-gu, Incheon 406-840, Korea; sjh11@yonsei.ac.kr

**Keywords:** *Houttuynia cordata*, quercitrin, solid lipid nanoparticles, sustained release, poloxamer

## Abstract

*Houttuynia cordata* (*H. cordata*) has been used for diuresis and detoxification in folk medicine as well as a herbal medicine with antiviral and antibacterial activities. *H. cordata* extract-loaded solid lipid nanoparticles (H-SLNs) were prepared with various concentration of poloxamer 188 or poloxamer 407 by a hot homogenization and ultrasonication method. H-SLNs dispersion was freeze-dried with or without trehalose as a cryoprotectant. The physicochemical characteristics of H-SLNs were evaluated by dynamic laser scattering (DLS), differential scanning calorimetry (DSC), Fourier transform infrared spectroscopy (FT-IR), and scanning electron microscopy (SEM). Additionally, the in vitro release and in vitro cytotoxicity of H-SLNs were measured. Encapsulation efficiencies of H-SLNs (as quercitrin) were 92.9–95.9%. The SEM images of H-SLNs showed that H-SLNs have a spherical morphology. DSC and FT-IR showed that there were no interactions between ingredients. The increased extent of particle size of freeze-dried H-SLNs with trehalose was significantly lower than that of H-SLNs without trehalose. H-SLNs provided sustained release of quercitrin from *H. cordata* extracts. Cell viability of Caco-2 cells was over 70% according to the concentration of various formulation. Therefore, it was suggested that SLNs could be good carrier for administering *H. cordata* extracts.

## 1. Introduction

*Houttuynia cordata* (*H. cordata*) is a traditional Chinese medicine used for hundreds of years to relieve lung-related symptoms such as lung abscesses, phlegm, coughs and dyspnea [1]. It is well known that *H. cordata* is rich in essential oils, alkaloids, and flavonoids. The flavonoids such as quercitrin, isoquercitrin, and rutin have been found to possess inhibitory effects on allergies, leukemia, oxidation, mutagenesis, hypertension, and inflammation [2]. Recently, several studies have provided scientific data to support its anti-inflammatory, anti-allergic, anti-oxidative, and anti-cancer activities [3].

There were some studies on ethanol extracts of *H. cordata* [4]. However, it was necessary to prepare the proper solid formulation of *H. cordata* extracts because *H. cordata* ethanol extracts were viscous and needed taste masking. In addition, quercitrin, an active molecule of *H. cordata,* is known to be poorly water-soluble (~1.8 mg/mL), which may cause low oral bioavailability. Solid lipid nanoparticles (SLNs) consist of an active compound dispersed in a melted solid lipid or a mixture of solid lipids, whereby the active molecules are incorporated between fatty acid chains when the lipid matrix was cooled down. SLNs have been known to have diverse advantages such as controlled drug release, drug targeting, increased drug stability, improved oral bioavailability, incorporation of lipophilic as well as hydrophilic drugs, no toxicity, avoidance of organic solvents and no problems with respect to large-scale production and sterilization [5,6,7,8,9]. SLNs can be prepared by various preparation methods such as high-pressure homogenization, high shear homogenization, ultrasound, solvent emulsification/evaporation, microemulsion technique, solvent emulsification/diffusion, double emulsion technique, membrane contactor technique and the supercritical fluid technique [10].

The aim of this study was to prepare and evaluate *H. cordata* extract-loaded SLNs (H-SLNs). Therefore, H-SLNs were prepared by a homogenization and sonication method [11,12]. The effects of the type and concentration of surfactants on the characteristics of H-SLNs, for example, encapsulation efficiency, particle size, FT-IR, DSC and surface morphology were observed. In addition, in vitro release profiles of quercitrin from H-SLNs and cytotoxicity of H-SLNs against Caco-2 cells were examined.

## 2. Results and Discussion

### 2.1. Particle Size, PDI and EE of H-SLNs

Table 1 shows the particle size, polydispersity index (PDI) and encapsulation efficiency (EE) of H-SLNs dispersion. F1–F6 were prepared with P188, and F7–F12 were prepared with P407 as a surfactant. It was found that the particle size of H-SLNs decreased as the concentration of surfactant was increased. This could be due to the significant reduction in the interfacial tension between organic and aqueous phase, which leads to a more homogenized lipid in the aqueous phase and the reduction of the particle size of SLNs [9,13]. Also, at high surfactant concentration, surfactant molecules sufficiently cover the lipid matrix leading to more stabilized particles.

It was observed that the prepared SLNs were less than 500 nm in size and increase in the surfactant concentration led to decrease in particle size of all SLN formulations [14]. On the other hand, there is a report that relatively high concentrations of surfactants are needed to prevent particle aggregation [15].

**Figure 1:**
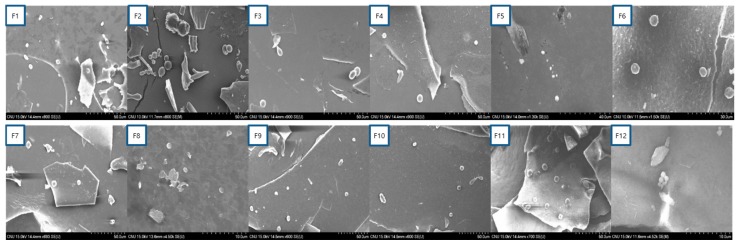
Scanning electronic microscopy images of various H-SLNs.

Figure 1 shows the morphologies of the various H-SLNs. All of the formulations were observed to be spherical in shape.

**Figure 2:**
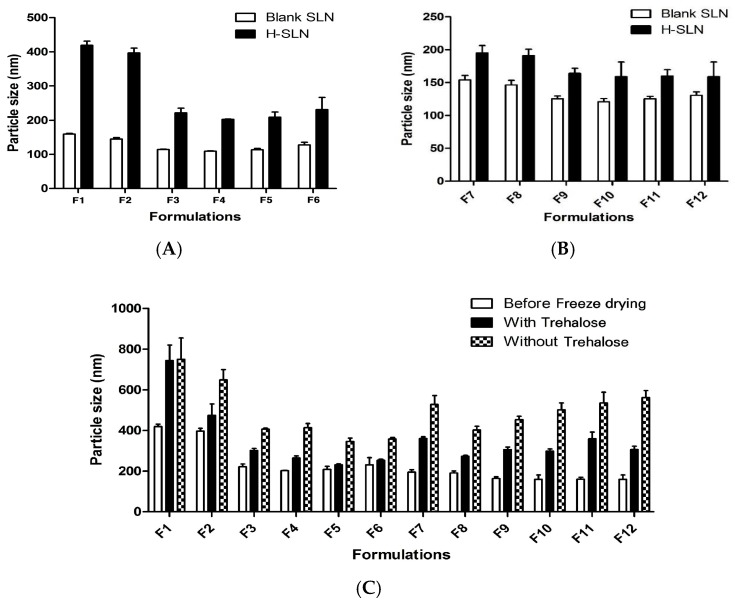
Effect of surfactant concentration in SLNs on the particle size. (**A**) Poloxamer 188 tailored SLNs; (**B**) Poloxamer 407 tailored SLNs. (**C**) Effect of the use of trehalose as a surfactant on the particle size. Data are expressed as the mean ± S.D. (*n* = 3).

The particle size of blank-SLNs was compared with the particle size of H-SLNs (Figure 2). The particle size of SLNs was increased with the incorporation of *H. cordata* extracts (Figure 2A,B). Particularly, the particle size decreases with increasing the concentration of P188 from 0.5% to 2%, but the particle size was increased by 2% or more, which was consistent with [16]. Also, the particle size of H-SLNs prepared using P407 was smaller than that of H-SLNs prepared with P188, which was consistent with [17]. It was suggested that a reduced diffusion rate of the solute molecules caused by an increased viscosity of the outer phase might be responsible for the particle size shift. Also, poloxamer was observed to show flocculation that may be attributed to the dehydration of the poloxamer chains and reduced steric stabilization efficiency at the increased concentration [16].

Generally, trehalose was proved to be a very effective cryoprotectant for SLN [18,19,20]. Trehalose is a disaccharide formed by a α,α-1,1-glucoside bond between two α-glucose units. Not only does this sugar have the advantage of protecting cells from disruption, but can also act as an antioxidant. It is used as a cryoprotectant for nanoparticles, because of its ability to preserve their original size and structure after freeze-drying [21].

The effect of trehalose (5%) on the H-SLNs during freeze-drying was evaluated by measuring the particle size (Figure 2C). The particle size of freeze-dried H-SLNs was generally increased compared with before freeze-drying. However, the extent of the particle size increase of freeze-dried H-SLNs with trehalose was significantly lower than that of H-SLNs without trehalose. When the nanoparticle dispersion was freeze-dried with cryoprotectant, the cryoprotectants form a glassy/vitreous coating around the nanoparticles protecting them against stresses like the mechanical stress of ice crystals, thereby preventing aggregation [22].

The EE of *H. cordata* extracts in H-SLNs was measured by analyzing the quercitrin which is the main component of *H. cordata*. It was confirmed that EE of *H. cordata* extracts in all formulations was over 90%, strongly indicating the great loading ability of the formulations (Table 1).

### 2.2. DSC Analysis of H-SLNs

**Figure 3:**
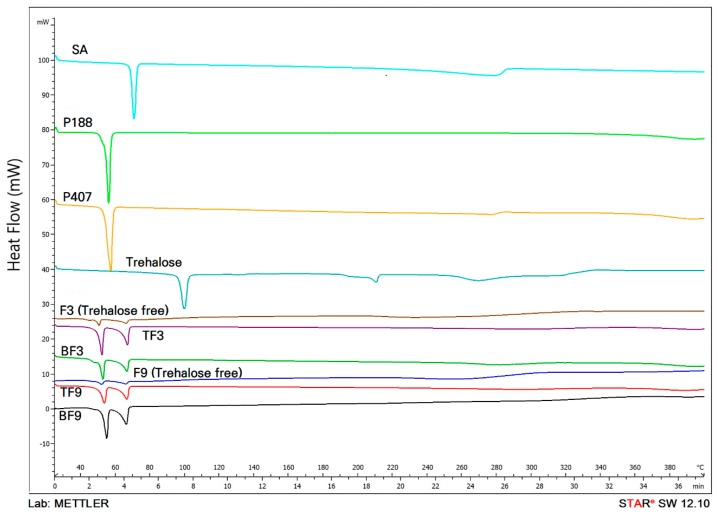
DSC thermograms of stearic acid (SA), poloxamer 188 (P188), poloxamer 407 (P407), trehalose, blank F3 (BF3), F3 (Trehalose free), trehalose added F3 (TF3), blank F9 (BF9), F9 (Trehalose free) and trehalose added F9 (TF9).

Formulation development of SLNs aims at ensuring physical stability in both particle size and the crystalline state of the lipid matrix. Therefore, it is necessary to prove the solid state of the lipid in SLNs by DSC analysis [23]. DSC thermograms of SA, P188, P407, blank F3, blank F9, F3 and F9 are shown in Figure 3.

The DSC peaks showed a broad peak without a specific maximum. The thermograms of SA, P188, and P407 showed endothermic peaks at 70.24, 55.47 and 56.79 °C, respectively. The thermogram of F3 exhibited peaks related to SA and P188. When compared with the thermogram of blank F3, thermogram of F3 did not have significant changes. The thermogram of F9 displays the peaks of SA and P407. The slight displacements of the SA and surfactant peaks in F3 and F9 indicated new molecular arrangements produced by the chemical interactions among the ingredients [24].

### 2.3. FT-IR Analysis of H-SLNs

**Figure 4:**
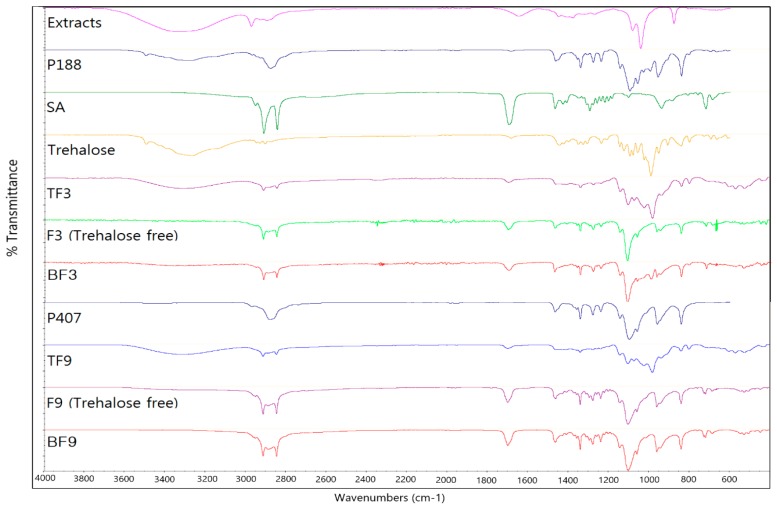
FT-IR spectra of *H. cordata* extracts, stearic acid (SA), poloxamer 188 (P188), poloxamer 407 (P407), trehalose, blank F3 (BF3), F3 (trehalose free), trehalose added F3 (TF3), blank F9 (BF9), F9 (trehalose free) and trehalose added F9 (TF9).

Figure 4 shows the FT-IR spectra of H-SLNs and their ingredients. The FT-IR spectrum of SA showed strong peaks at 1698 cm^−1^ (C=O stretching) and 2847 cm^−1^ (CH stretching). The long-chain bond is seen in 720 cm^−1^ and the CH_2_ and CH_3_ bands are seen in around 1550 cm^−1^ and 1300 cm^−1^, respectively. The FT-IR spectrum of F3 and F9 with trehalose (TF3 and TF9) shows approximately all peaks of SA with some minor displacements for 2913 cm^−1^ and 2849 cm^−1^ (CH stretching) which is related to re-arrangement of molecules in the SLN structure. Also, in the TF3 and TF9 spectra, an additional peak at 1098 cm^−1^ is related to the P188 and P407 and the peaks at around 3311 cm^−1^ and 990 cm^−1^ are related to trehalose. In the F3 and F9 without trehalose, the peaks related to the trehalose was not observed.

### 2.4. In Vitro Release Study

**Figure 5:**
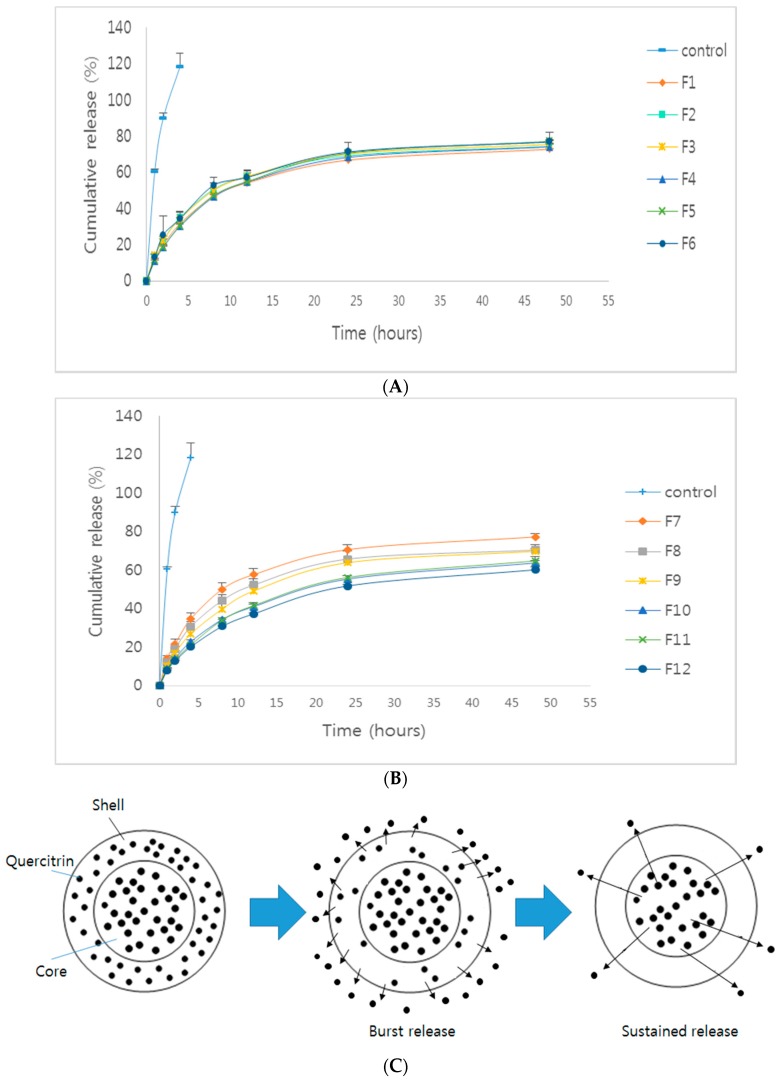
In vitro release of all formulations (1% tween 80, pH 7.4). (**A**) F1–F6; (**B**) F7–F12 and (**C**) Schematic drawing of the proposed release mechanism of drug from solid lipid nanoparticles.

For observing the effect of type and concentration of surfactants on the release profile of quercitrin from H-SLNs, in vitro release studies using all formulations were conducted. *H. cordata* extracts released 100% of quercitrin within 4 h. Release patterns of quercitrin from F1–F12 with an initial burst release were observed, followed by a sustained release of quercitrin (Figure 5). The magnitude of the burst release has a direct relation to the amount of quercitrin existing on the surface of H-SLNs [9,25,26]. The sustained release was due to the release of quercitrin from the lipid matrix core [27]. Also, the release rate of quercitrin from H-SLNs with P407 (F7–F12) was more sustained according to the concentration of P407. These results suggested that the amount of quercitrin in the lipid matrix core was increased by increasing the concentration of P407. However, there is a discrepant publication reporting that formulations showed an increase in the percentage drug release with the increase in the surfactant concentration. This can be attributed to the solubilization effect of the surfactants. The increase in the surfactant concentration helps the drug go into solution [28].

Usually, three kinds of drug-incorporation model are considered for SLNs, including the solid solution model, drug-enriched shell model and drug-enriched core model [25,26]. The solid solution model SLNs can be prepared by the cold homogenization method. The drug is rapidly released from SLNs in the drug-enriched shell model. In addition, drug-enriched core model does not show the initial burst release. Therefore, drug incorporation model of *H. cordata* extracts in H-SLNs can be assumed as a combined model of drug-enriched core model and drug-enriched shell model (Figure 4C).

### 2.5. In Vitro Cytotoxicity Study

**Figure 6:**
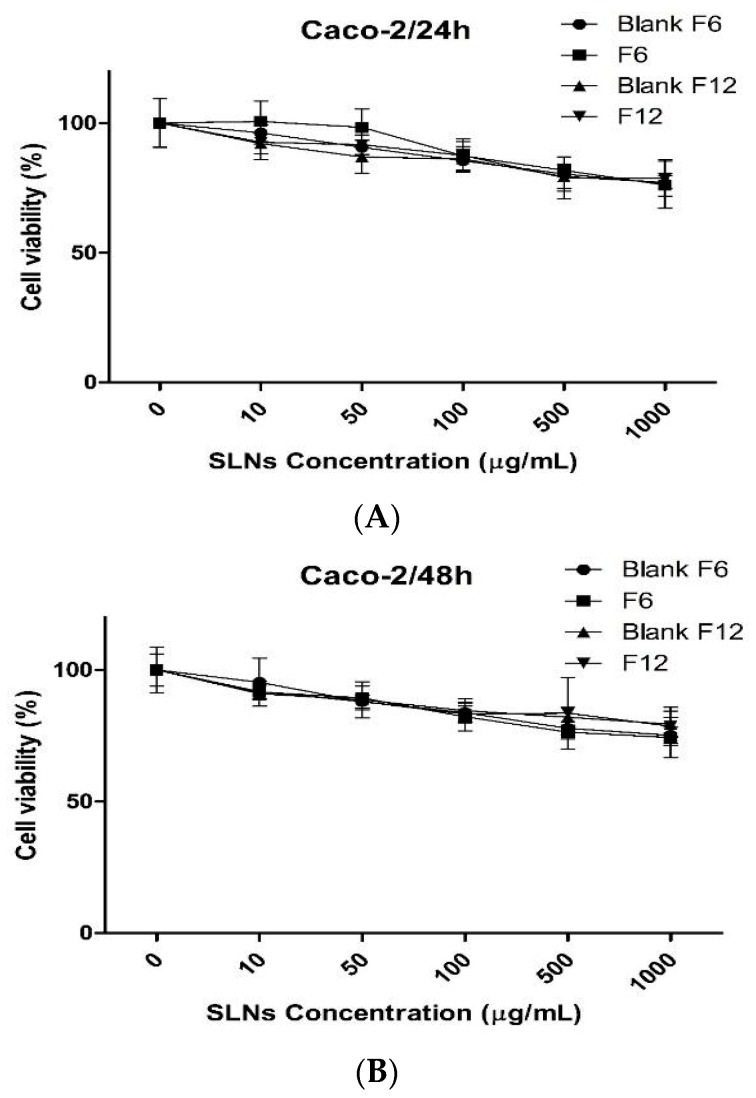
Cell viability of Caco-2 cells treated with blank F6, F6, blank F12 and F12 for (**A**) 24 and (**B**) 48 h (*n* = 3).

In terms of in vitro cell studies, the types of cells are important in understanding cellular mechanisms related to physiological conditions in humans. In particular, the Caco-2 cells are commonly used as representing models of the human intestinal epithelium [29,30]. Caco-2 cells are known to mimic typical properties of the human small intestinal epithelium such as a well-developed brush border with associated enzymes such as alkaline phosphatase and sucrose isomaltase [31]. Therefore, we investigated the toxicity of different concentrations of H-SLNs using MTT assay as an indicator of cytotoxic effects (Figure 6). For 24 h or 48 h incubation, cell viability of Caco-2 cells was over 70% according to the concentration of various formulation. Even the concentration of the formulations was treated up to 1000 µg/mL, the cell viability did not drop down below 70%. It has been known that cell viability >70% is considered as “no toxicity” [32]. Overall, the results obtained suggested that the composition of H-SLNs did not possess severe toxicity for oral delivery purposes.

## 3. Materials and Methods

### 3.1. Materials

Stearic acid (SA) was purchased from Daejung Chemical (Cheongwon, Korea). Poloxamer 188 (P188) and poloxamer 407 (P407) were obtained from BASF (Ludwigshafen, Germany). Quercitrin was purchased from Sigma-Aldrich (Steinheim, Switzerland). D-(+)-Trehalose dehydrate was purchased from Acros Organics (Pittsburgh, PA, USA). All other chemicals were commercial products of analytical or reagent grade and used without further purification.

### 3.2. Cell Cultures

Caco-2 cells (human colon adenocarcinoma cells) was purchased from Korean Cell Line Bank (Seoul, Korea). Caco-2 cells were cultured with minimum essential medium (MEM) supplemented with 10% fetal bovine serum (FBS) and 100 units/mL penicillin (GIBCO BRL, Grand Island, NY, USA), respectively, in a humidified atmosphere of 5% CO_2_ at 37 °C.

### 3.3. Extraction of H. cordata

*H. cordata* extract was obtained from Wissen Co. (Daejeon, Korea). Briefly, 4 L of 95% ethanol was added to 200 g of dried *H. cordata* raw material. This mixture was sonicated (30 min running and 30 min stop cycle, 10 cycles). This extract was filtered with 5 µm-filter paper and 1 µm-filter paper, successively. Extracts of *H. cordata* were stored in the refrigerator until use.

### 3.4. Preparation of H-SLNs

H-SLNs were prepared by homogenization and ultra-sonication method. Briefly, 200 μL of *H. cordata* extracts was added to 200 mg of SA and then the mixtures were heated to 75 °C. Separately, 0.5–3% of P188 or P407 was heated to 75 °C. The heated surfactant solution was added to the *H. cordata*-lipid melted mixture. This solution was homogenized at 12,000 rpm for 3 min, subsequently sonicated at 80 °C for 10 min in water bath. H-SLNs dispersion was formed by cooling this pre-emulsion at 4 °C. H-SLNs dispersion was freeze-dried with or without trehalose as a cryoprotectant. As well as, blank SLNs were prepared without *H. cordata* extracts as above protocol and freeze-dried with trehalose. Table 1 shows the compositions for the preparation of H-SLNs. Morphology of the H-SLNs was characterized by SEM. The nanoparticles were mounted on aluminium stubs, sputter-coated with a thin layer of Au/Pd, and examined by using an SEM (JSM-7000F, JEOL, Tokyo, Japan).

### 3.5. Measurement of Particle Size and Polydispersity Index (PDI) of H-SLNs

The particle size as well as PDI of H-SLNs dispersion and lyophilized H-SLNs was measured by dynamic light scattering (DLS) using a Zetasizer Nano ZS (Malvern Instruments, Malvern, UK). For measuring the particle size, H-SLNs dispersion was diluted to 10 times with distilled water and 10 mg of lyophilized H-SLNs were dispersed in distilled water.

### 3.6. Encapsulation Efficiency (EE) of H. cordata Extracts in H-SLNs

Quercitrin is the main component of *H. cordata*. The EE of quercitrin from H-SLNs was measured as follows. Briefly, 0.5 mL of H-SLNs dispersion was diluted with 1 mL of ethanol and heated at 75 °C for 20 min. After cooling at room temperature, this solution was centrifuged at 3000 rpm for 5 min to precipitate the undissolved solid lipid, filtered through a 0.45 μm syringe filter and injected into the HPLC system with following conditions; an Agilent 1100 liquid chromatography system with an autosampler and UV detector was used with a C18 column (4.6 × 250 mm, 5 µm particle size, Agilent, Santa Clara, CA, USA). The flow rate was 1 mL/min, and the detection wavelength was set at 254 nm. The mobile phase A was 2% acetic acid solution in distilled water and B was acetonitrile. EE was calculated applying the following formula: EE (%) = (amount of quercitrin in H-SLNs/amount of the feeding quercitrin) × 100.

### 3.7. Differential Scanning Calorimeter (DSC) Analysis of H-SLNs

In order to assess the change of solid state, DSC analysis was performed on freeze-dried H-SLNs with trehalose. To figure out the thermal behavior of freeze-dried H-SLNs with trehalose, blank F3, F3 (freeze-dried H-SLNs without trehalose), TF3 (with trehalose), blank F9, F9 (freeze-dried H-SLNs without trehalose), and TF9 (with trehalose) were compared as well as the components such as *H. cordata* extracts, SA, P188, P407 and trehalose. Accurately weighed samples of 2 mg were analyzed in aluminium pans on a DSC (DSC S-650, Scinco, Daejeon, Korea). The DSC runs were conducted from 10 to 400 °C.

### 3.8. Fourier Transform Infrared (FT-IR) Analysis of H-SLNs

In order to assess the interactions between ingredients of freeze-dried H-SLNs, FT-IR analysis using a Thermo Nicolet 380 spectrophotometer (Thermo Scientific, Waltham, MA, USA) was performed on freeze-dried H-SLNs with trehalose. To figure out the interactions of freeze-dried H-SLNs with trehalose, blank F3, F3 (freeze-dried H-SLNs without trehalose), TF3 (with trehalose), blank F9, F9 (freeze-dried H-SLNs without trehalose), and TF9 (with trehalose) were compared as well as the components such as *H. cordata* extracts, SA, P188, P407 and trehalose. FT-IR was conducted and then, the spectrum was recorded in the wavelength number of 4000 to 500 cm^−1^.

### 3.9. In Vitro Release Study

In vitro release study of quercitrin from H-SLNs was evaluated using a dialysis bag (Spectra/Por Cellulose Ester Membrane MWCO: 25 kDa, Spectrum Labs, Rancho Dominguez, CA, USA), which was filled with 2 mL of 1% tween 80 (pH 7.4). The dialysis bag was immersed in 5 mL of 1% tween 80, then it was stirred at 100 rpm in a shaking bath at room temperature. At predetermined time intervals (1, 2, 4, 8, 12, 24 and 48 h), 2 mL of samples was withdrawn from the medium and replaced with the same volume of fresh medium. The obtained samples were filtered with 0.45 µm syringe filter and the amount of quercitrin was determined by HPLC. Percent of cumulative release at each time was normalized to the total amount of quercitrin in the tube. All samples were run in triplicate and data points are shown as a mean ± standard deviation.

### 3.10. In Vitro Cytotoxicity Study

The cytotoxicity of F6 and F12 was assessed using the Caco-2 cells by a MTT kit (Sigma-Aldrich). The Caco-2 cells were seeded in 96-well plates at a cell density of 5 × 104 cells/mL (200 µL/well). The cells were further incubated for 24 h or 48 h in fresh culture media containing blank F6, blank F12, F6 and F12 with various concentrations. The culture medium was replaced by MTT solution (5 mg/mL, 100 µL/well), followed by incubation for a further 3 h. The MTT solution was removed and 200 µL of dimethyl sulfoxide was added to the wells for dissolving formazan. The plates were then placed in an incubator for 30 min. The absorbance values of each well were recorded at 570 nm using a microplate reader (Sunrise; Tecan, Austria GmbH, Grodig, Austria).

### 3.11. Statistical Analysis

The student’s *t*-test was used to compare two different groups of samples. A *p*-value < 0.05 was considered significant.

## 4. Conclusions

In this study, we prepared H-SLNs by a homogenization and ultrasonication method using various concentrations of P188 or P407. We confirmed that surfactant concentration affects the particle size of H-SLNs. H-SLNs have a high EE, a spherical shape with a smooth surface, and a sustained release profile. The cell viability of Caco-2 cells indicated the safety of H-SLNs as oral drug delivery systems. Overall, SLNs tailored by P407 may be a promising delivery platform for oral delivery of *H. cordata* extracts.

## Figures and Tables

**Table 1:** Formulations and the particle size, PDI and EE of H-SLNs dispersion; Data are expressed as the mean ± S.D. (*n* = 3). The volume of surfactant solution and *H. cordata* extracts is 20 mL and 0.2 mL, respectively.

**Formulation**	**Poloxamer 188 (%)**	**Particle Size (nm)**	**PDI**	**EE (%)**
F1	0.5	419.1 ± 12.0	0.060 ± 0.035	91.3 ± 1.4
F2	1	396.6 ± 14.2	0.184 ± 0.011	89.2 ± 2.3
F3	1.5	221.4 ± 13.7	0.232 ± 0.011	93.1 ± 4.6
F4	2	202.9 ± 1.1	0.216 ± 0.014	91.2 ± 4.4
F5	2.5	209.0 ± 14.8	0.206 ± 0.23	93.0 ± 2.6
F6	3	231.0 ± 35.6	0.223 ± 0.27	94.3 ± 2.5
**Formulation**	**Poloxamer 407 (%)**	**Particle Size (nm)**	**PDI**	**EE (%)**
F7	0.5	195.2 ± 11.4	0.096 ± 0.037	96.3 ± 2.3
F8	1	191.3 ± 9.6	0.154 ± 0.021	93.3 ± 3.6
F9	1.5	164.1 ± 7.7	0.169 ± 0.012	97.0 ± 4.0
F10	2	159.6 ± 22.3	0.166 ± 0.013	91.3 ± 5.1
F11	2.5	160.7 ± 9.7	0.087 ± 0.010	92.8 ± 2.0
F12	3	159.6 ± 22.3	0.233 ± 0.015	95.5 ± 2.2
